# Brown Adipose Tissue, Diet-Induced Thermogenesis, and Thermogenic Food Ingredients: From Mice to Men

**DOI:** 10.3389/fendo.2020.00222

**Published:** 2020-04-21

**Authors:** Masayuki Saito, Mami Matsushita, Takeshi Yoneshiro, Yuko Okamatsu-Ogura

**Affiliations:** ^1^Faculty of Veterinary Medicine, Hokkaido University, Sapporo, Japan; ^2^Department of Nutrition, Tenshi College, Sapporo, Japan; ^3^Division of Metabolic Medicine, Research Center for Advanced Science and Technology, The University of Tokyo, Tokyo, Japan

**Keywords:** bile acids, brown adipose tissue, diet-induced thermogenesis, food ingredients, gut hormone, obesity, sympathetic nervous system, transient receptor potential channels

## Abstract

Since the recent rediscovery of brown adipose tissue (BAT) in adult humans, this thermogenic tissue has been attracting increasing interest. The inverse relationship between BAT activity and body fatness suggests that BAT, because of its energy dissipating activity, is protective against body fat accumulation. Cold exposure activates and recruits BAT, resulting in increased energy expenditure and decreased body fatness. The stimulatory effects of cold exposure are mediated through transient receptor potential (TRP) channels and the sympathetic nervous system (SNS). Most TRP members also function as chemesthetic receptors for various food ingredients, and indeed, agonists of TRP vanilloid 1 such as capsaicin and its analog capsinoids mimic the effects of cold exposure to decrease body fatness through the activation and recruitment of BAT. The antiobesity effect of other food ingredients including tea catechins may be attributable, at least in part, to the activation of the TRP–SNS–BAT axis. BAT is also involved in the facultative thermogenesis induced by meal intake, referred to as diet-induced thermogenesis (DIT), which is a significant component of the total energy expenditure in our daily lives. Emerging evidence suggests a crucial role for the SNS in BAT-associated DIT, particularly during the early phase, but several gut-derived humoral factors may also participate in meal-induced BAT activation. One intriguing factor is bile acids, which activate BAT directly through Takeda G-protein receptor 5 (TGR5) in brown adipocytes. Given the apparent beneficial effects of some TRP agonists and bile acids on whole-body substrate and energy metabolism, the TRP/TGR5–BAT axis represents a promising target for combating obesity and related metabolic disorders in humans.

## Introduction

Brown adipose tissue (BAT) has long been recognized as the major site of non-shivering thermogenesis (NST) during cold exposure [cold-induced thermogenesis (CIT)] and arousal from hibernation in small rodents (1). Since the rediscovery of metabolically active BAT using fluorodeoxyglucose (FDG)-positron emission tomography (PET) and computed tomography (CT) in adult humans (2–5), subsequent experimental and clinical studies have dramatically increased our knowledge about the pathophysiological roles of BAT in the regulation of energy balance and body fatness (6, 7). Human BAT, as in the case of rodent BAT, is activated by acute cold exposure (2, 5) or administration of β-adrenergic receptor (βAR) agonists (8), and contributes to increasing whole-body energy expenditure (EE) and fatty acid oxidation (9–12). The activity and prevalence of BAT substantially decrease in older and obese populations (2, 3, 13–16), and this age-related decline in BAT activity is closely associated with visceral fat accumulation (17). Prolonged exposure to cold recruits BAT, increases EE, and decreases body fat content (18–20). In addition, cold exposure improves glucose metabolism and insulin sensitivity (21–24). Thus, BAT could be a promising target to boost whole-body EE and prevent obesity and related metabolic disorders in humans (25–30).

Although cold exposure is undoubtedly the most physiological and effective regimen to activate and recruit BAT, it would be difficult and uncomfortable to increase human exposure to cold temperatures under well-controlled conditions with the presence of clothing and heating systems. Moreover, chronic cold exposure increases blood pressure (8) and may induce atherosclerosis (31). Thermogenesis observed after food intake [diet-induced thermogenesis (DIT)] is another component of NST. Although the involvement of BAT in DIT has been demonstrated in small rodents, only limited information is currently available in humans. The aim of this review article is to summarize and discuss the evidence for a role of BAT in DIT and thermogenesis induced by certain food ingredients in humans, considering that DIT is a significant component of whole-body EE in our usual daily life.

## Cold-Induced Bat Thermogenesis

Although the mechanism of BAT-dependent CIT has mostly been investigated in small rodents, essentially the same mechanism is believed to work in humans. When animals are exposed to cold temperatures, cold is perceived by temperature sensors, transient receptor potential (TRP) channels, which are membrane proteins that transmit information about changes in the environment such as temperature, touch, pain, osmolarity, and naturally occurring substances (32). Cold-activated TRP on sensory neurons on the body surface transmit information to the brain and increase the activity of sympathetic nerves entering BAT (33). Noradrenaline (NA) released from sympathetic nerve endings stimulates brown adipocytes via the βAR and triggers cyclic adenosine monophosphate (cAMP)-activated intracellular events including hydrolysis of triglyceride, oxidation of resulting fatty acids, and activation of uncoupling protein 1 (UCP1), a key mitochondrial molecule for BAT thermogenesis. Sympathetic activation also results in increased fat mobilization in white adipose tissue, and released fatty acids are used in peripheral tissues including BAT. Although the principal substrate for BAT thermogenesis is fatty acids, glucose utilization is also enhanced in parallel with UCP1 activation, probably for a sufficient supply of oxaloacetate to enable the rapid oxidation of fatty acids and acetyl coenzyme A (CoA), and also for recovery of cellular adenosine triphosphate (ATP) levels by activating anaerobic glycolysis (34). Thus, UCP1-dependent glucose utilization could be a metabolic index of BAT thermogenesis, and has been applied in FDG-PET for assessing human BAT.

When animals are exposed to cold temperatures for a long time, they adapt to their surroundings by increasing the number of brown adipocytes and the amount of UCP1 through the proliferation of interstitial preadipocytes and matured adipocytes (35, 36). In addition to BAT hyperplasia, prolonged cold exposure gives rise to an apparent induction of UCP1-positive adipocytes in white adipose tissue. This type of adipocytes, termed “beige” or “brite” cells, is developmentally distinct from “classical” brown adipocytes, which derive from Myf5-positive myoblastic cells (6, 37). Thus, chronic cold exposure results in increased EE through the persistent activation and recruitment of classical brown adipocytes and beige cells, and the consequent “browning” of white adipose tissue and body fat reduction. As the FDG-PET/ CT-detected and UCP1-positive human adipose depot consists of a mixture of brown and beige adipocytes (38–41), hereafter we shall refer to it collectively as BAT.

## Diet-Induced Activation of Bat

EE above the basal metabolic rate in response to meal intake is referred to as the “specific dynamic action of food,” “thermic effects of food,” or “DIT.” The term DIT has often been used to describe the adaptive increase in EE observed after long-term overfeeding, which is also known as “luxury consumption” or “luxosconsumption.” Since the publication of the report of Rothwell and Stock (42) in 1979, it has been demonstrated in small rodents that daily spontaneous feeding of high-calorie diets such as high-fat and cafeteria diets resulted in a lower energy efficiency with less weight gained than expected on the basis of on caloric intake, in parallel with an increased BAT activity and EE (43). The adaptive changes in response to overfeeding are not observed in animals without UCP1 (44). Thus, the role of BAT in adaptive increase in EE and maintaining energy balance seems to have been accepted, albeit not widely supported (45).

Thermogenesis after a single meal is expressed as the percentage of the energy content of the food ingested (~10% for standard meals in humans). This is usually divided into two components: obligatory and facultative thermogenesis. Obligatory thermogenesis refers to the obligatory response including digestion, absorption, and storage of ingested nutrients, whereas facultative thermogenesis refers to the additional responses to obligatory thermogenesis and may be closely related to the adaptive increase in EE. In this work, we tentatively refer to facultative thermogenesis in response to single meals as DIT and discuss its mechanisms and pathophysiological relevance in the context with BAT.

Activation of BAT after a single meal was suggested in the 1980s by Glick et al. (46), who reported increased respiration rate of BAT in 2 h after food intake in rats. They also demonstrated a meal-induced increase in guanosine 5′-diphosphate (GDP) binding to mitochondria isolated from BAT, which was used as an index of UCP1 activation (47). Our team (48) found meal-induced metabolic activation of BAT in rats—that is, in 30 min after oral intake of a liquid meal, glucose utilization and fatty acid synthesis were increased in intact BAT, but to a much lower extent in surgically denervated BAT. The critical role of UCP1 in DIT was proved by simultaneous 24-h recording of food intake and oxygen consumption in UCP1-deficient mice maintained at a thermoneutral temperature of 30°C—that is, whole-body oxygen consumption in UCP1-deficient mice was lower than that in wild-type mice, particularly during the eating period (44).

## Diet-Induced Bat Thermogenesis in Humans

In humans, the possible contribution of BAT thermogenesis to DIT and regulation of energy balance have been suggested by studies on single nucleotide polymorphism in some BAT-related genes. For example, Trp64Arg mutation in the β3 adrenergic receptor (β*3AR*) gene and A3826G mutation in the *UCP1* gene are associated with higher body fatness, lower metabolic rate, and smaller weight loss via treatment with low-calorie diets (49–52). Nagai et al. (53) examined the effects of A-3826G mutation in the *UCP1* gene on DIT in boys, and found a reduced response 3 h after a high-fat meal in those carrying the G allele. They also found diminished CIT in the group with the GG allele compared with those carrying the AA + AG alleles (54).

Rediscovery of BAT in adult humans has prompted further studies to test whether BAT thermogenesis is activated after single meals. Vrieze et al. (55) measured BAT activity using FDG-PET/CT in healthy volunteers 90 min after meal intake, and unexpectedly found a reduction in FDG uptake into BAT compared with that after overnight fasting. Vosselman et al. (56) also reported that postprandial FDG uptake into BAT was much lower than cold-induced uptake, whereas whole-body EE was comparable. Although these results seem to be in conflict with the idea of postprandial activation of BAT thermogenesis, they can be explained by increased insulin-stimulated FDG uptake into skeletal muscle, which reduces FDG bioavailability for BAT, which in turn leads to underestimation of BAT activity. FDG uptake after a mild cold exposure is increased specifically in BAT, whereas that after food intake is increased in many insulin-sensitive tissues such as skeletal muscle, brown and white adipose tissue, and heart (10). Thus, although FDG uptake into BAT can be used as an index of BAT activity under certain restricted conditions, it is not always associated with BAT thermogenesis.

This limitation of FDG-PET/CT is overcome by measuring oxygen uptake using ^15^O[O_2_]-PET and blood flow using ^15^O[H_2_O]-PET, which are more descriptive indicators of thermogenesis and mitochondrial substrate oxidation. In fact, acute cold exposure evoked a marked increase in oxygen consumption and blood flow in parallel with increased whole-body EE (57). Moreover, Din et al. (58) demonstrated that oxygen consumption and blood flow in BAT rose immediately after meal intake to an extent comparable to those observed after cold exposure. To confirm the role of BAT in DIT, we measured whole-body EE continuously for 24 h in healthy humans using a human calorimeter (59). When the participants were divided into high BAT and low BAT groups according to the result of FDG-PET/CT examination, there was no significant difference in body composition and resting EE between the two groups. However, EE after meals was significantly higher in the high BAT group (9.7% of the total energy intake) than in the low BAT group (6.5%). Of note, the 24-h respiratory quotient was also apparently lower in the high BAT group, implying higher fat oxidation. Higher postprandial whole-body EE and substrate oxidation were also confirmed in participants with higher BAT activities (57). All these results indicate that BAT contributes to DIT, at least in part, in humans. This may also be indirectly supported by the finding that BAT recruitment by prolonged cold exposure is accompanied by enhanced DIT (21).

## Mechanisms of DIT: the Sympathetic Nervous System (SNS)

**Figure 1:**
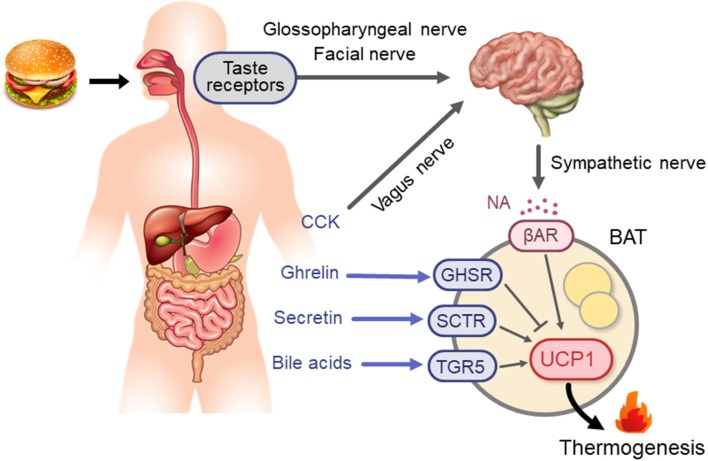
Neural and endocrine mechanisms for BAT thermogenesis activated after meal intake. βAR, β-adrenergic receptor; BA, bile acids; BAT, brown adipose tissue; CCK, cholecystokinin; GHSR, ghrelin receptor; NA, noradrenaline; SCTR, secretin receptor; TGR5, G-protein-coupled bile acid-activated receptor; UCP1, uncoupling protein 1.

Based on the principal role of the SNS–βAR axis for CIT, it is conceivable that this axis is a key mechanism in diet-induced/postprandial BAT thermogenesis (Figure 1). In fact, in both experimental animals and humans, the plasma levels of NA and tissue NA turnover are low during fasting but increases immediately after food intake (60–63). Moreover, SNS activity in BAT estimated from tissue NA turnover is increased in mice chronically overfed with cafeteria and high-calorie diets (64, 65). Meanwhile, our team (48) found that in rats metabolic activation of BAT after intake of a liquid meal was diminished after surgical severing of sympathetic nerves entering BAT. These results are in line with the idea that diet-induced/postprandial BAT thermogenesis is mediated through sympathetic nerve activation. One interesting observation in our studies was that the meal-induced metabolic activation and NA turnover in BAT were reduced in rats given the same meal through a gastric tube. In the case of humans and dogs, LeBlanc et al. (66, 67) showed that responses in oxygen consumption, and plasma levels of NA and insulin shortly (1–2 h) after food intake were substantially reduced when food was administered through a stomach tube. They also reported lowered postprandial thermogenesis with a non-palatable meal in comparison with a highly palatable meal, despite using the same composition and amount in both meals (68). These results suggest that food palatability and oropharyngeal taste sensation play a significant role in diet-induced sympathetic activation and BAT thermogenesis. This may be consistent with the observation that the cafeteria feeding regimen with palatable foods is most efficient in producing a concomitant voluntary hyperphagia, elevated SNS activity, and BAT hyperplasia, thereby resulting in “luxsusconsumption.”

Thus, the SNS–BAT axis may be crucial for DIT, particularly during the early phase, in the same way as for CIT; however, this implication still seems controversial in humans. Wijers et al. (69) reported considerable interindividual variations in thermogenic responses to 84-h intervention by overfeeding and mild cold exposure in 13 male individuals, but a high correlation between the responses to the two interventions, suggesting a common regulation mechanism shared in DIT and CIT. However, there have been reports of an apparent dissociation of DIT with CIT in cold-adapted humans. For example, Peterson et al. (70) demonstrated that daily exposure of healthy men to cold temperatures for 4 weeks resulted in a 2-fold increase in CIT, in parallel with increased SNS activity, whereas it did not change the thermic effect of food. Lee et al. (21) also reported a dissociation between the effects of prolonged cold exposure on DIT and CIT. Moreover, blockade of βAR with propranolol was demonstrated to have only a small effect on the increase in whole-body EE after intake of carbohydrate-rich meals (71–73). All these results suggest that DIT in humans is regulated by some mechanisms different from, and/or in combination with, the SNS–βAR axis.

## Mechanisms of DIT: Gut Hormones and Bile Acids

One of the likely factors involved in DIT may be gut hormones. Li et al. (74) found abundant expression of the secretin receptor (SCTR) in murine brown adipocytes, and demonstrated that secretin activates UCP1- and SCTR-dependent thermogenesis *in vitro* and *in vivo*. They also confirmed that the increment of plasma secretin levels induced by a single meal positively correlated with oxygen consumption and fatty acid uptake rates in BAT in humans. These observations collectively support the idea that meal-associated increase in circulating secretin activates BAT thermogenesis by binding to SCTR in brown adipocytes. Direct evidence for the thermogenic action of secretin on human BAT was obtained using FDG-PET/CT after secretin infusion, which significantly increased FDG uptake in supraclavicular BAT.

In addition to secretin, other gut hormones are also known to activate or suppress BAT thermogenesis in small rodents. Recently, Yamazaki et al. (75) reported that in rats, peripherally administered cholecystokinin (CCK) activates the SNS–BAT axis via the CCK receptor and vagal afferent nerves. Blouet and Shwartz (76) also demonstrated that in rats BAT thermogenesis induced by intraduodenal administration of lipids was abolished by administration of either the CCK receptor antagonist devazepide or the *N*-methyl-d-aspartate receptor blocker MK-801 directly into the caudomedial nucleus of the solitary tract. These results collectively indicate that CCK activates BAT thermogenesis via vagal afferent and sympathetic efferent nerves. In fact, Vijgen et al. (77) demonstrated that vagal afferents played a role in BAT thermogenesis in humans: vagus nerve stimulation significantly increases whole-body EE in parallel with BAT activity assessed by FDG-PET/CT.

CCK is an anorexigenic hormone secreted from the duodenum after food intake, whereas ghrelin is an orexigenic hormone and its secretion from the stomach is reduced after food intake. Lin et al. (78) reported that ghrelin decreases UCP1 expression in brown adipocytes, and that during aging, plasma ghrelin and ghrelin receptor expression in BAT increases whereas BAT thermogenesis declines. It is thus possible that reduced secretion of ghrelin, together with increased secretion of CCK and secretin, contributes to BAT activation in response to food intake. This may be supported by an association of BAT with systemic concentrations of some gut hormones including ghrelin in humans (79). Thus, there are multiple factors/mechanisms for diet-induced/postprandial BAT thermogenesis, their actions being synergistic or independent of each other. However, the precise nature of their roles in DIT and whole-body EE in humans remain largely unexplained to date.

Another humoral factor may be bile acids (BA), which are secreted into the intestinal lumen in response to meal intake, modified by gut flora, and mostly returned to the liver. During enterohepatic circulation, BA is partially transferred into general circulation, resulting in a rapid postprandial increase in its plasma concentration (80, 81). BA are now recognized as a metabolic regulator, affecting multiple functions, in addition to lipid-digestive functions, to regulate energy metabolism, as well as glucose and lipid metabolism, through the activation of nuclear farnesoid X receptor and the G protein-coupled membrane receptor TGR5 (Takeda G-protein receptor 5) (82). In connection with the thermogenic and antiobesity effects of BA, Watanabe et al. (83) demonstrated that in mice BA activates TGR5 in brown adipocytes, leading to activation of type 2 deiodinase and increased thermogenic activity. Similar direct stimulatory effects of BA chenodeoxycholic acid on BAT were reported in humans using brown adipocytes *in vitro* and using FDG-PET/CT *in vivo* (84). BA also stimulates intestinal L-cell TGR5 to secrete glucagon-like peptide-1 (GLP-1). Although GLP-1 is known as an incretin to stimulate insulin secretion, it activates BAT thermogenesis and induces beige fat development through the action on its receptor in the hypothalamus (85) and the AMPK–SIRT-1–PGC1-α (AMP-activated protein kinase–sirtuin 1–peroxisome proliferator-activated receptor gamma coactivator 1-alpha) cell signaling pathway (86). Crucial roles of TGR5 were also demonstrated in browning of white adipose tissue under multiple environmental cues including cold exposure and prolonged high-fat diet feeding (87).

## Bat Thermogenesis and Dietary Fat

**Figure 2:**
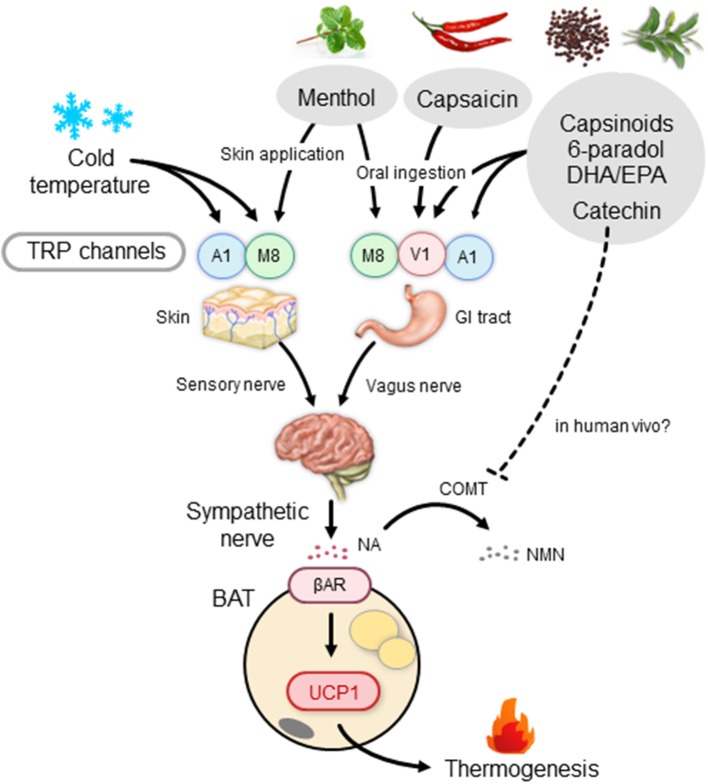
BAT thermogenesis through the activation of the TRP–SNS axis by food ingredients. βAR, β-adrenergic receptor; BAT, brown adipose tissue; COMT, catechol-*O*-methyl transferase; DHA, docosahexaenoic acid; EPA, eicosapentaenoic acid; GI tract, gastrointestinal tract; NA, noradrenaline; NMN, normetanephrine; SNS, sympathetic nervous system; TRP, transient receptor potential channel; A1, TRP ankyrin subfamily member 1; M8, TRP metastatin 8; V1, TRP vanilloid 1; UCP1, uncoupling protein 1.

Thermogenesis after a single meal is usually estimated to be 10% for standard meals; it varies depending on the composition of meals, being ~3% for fat, 7% for carbohydrate, and 30% for protein. In contrast, sympathetic and BAT activation, and probably facultative thermogenesis (DIT), are low in animals fed on high-protein diets (88, 89). Accordingly, high-fat diets and/or cafeteria diets with high carbohydrate and fat contents have been widely used for activation and recruitment of BAT. In this context, what is interesting is that some types of dietary fat including fish oil help prevent cardiovascular and metabolic diseases, and visceral fat accumulation (90). Moreover, several studies conducted in human volunteers have reported that postprandial thermogenesis is greater after intake of a meal rich in polyunsaturated fatty acids compared to that rich in monosaturated and saturated fatty acids (91, 92). Earlier studies in rats have revealed that dietary fish oil and/or eicosapentaenoic acid (EPA) and docosahexaenoic acid (DHA) rich in fish oil enhance EE and prevent fat accumulation by inducing UCP1 in BAT (93, 94). Kim et al. (95) reported that UCP1 induction by dietary EPA and DHA is blocked by either subdiaphragmatic vagotomy or treatment with a βAR blocker. They also demonstrated that the thermogenic and antiobesity effects of EPA and DHA are abolished in mice lacking TRP vanilloid 1 (TRPV1), a member of the TRP family activated by vanilloid compounds. Considering that EPA and DHA have agonistic activity on TRPV1, it is likely that these polyunsaturated fatty acids stimulate the vagus nerve through TRPV1 in the gut, thereby activating the SNS–βAR axis and BAT thermogenesis (Figure 2).

In addition, direct action mechanisms of EPA in brown adipocytes have also been proposed. To cite an example, Kim et al. (96) reported that EPA is sensed by the membrane receptor free fatty acid receptor 4 in brown adipocytes, resulting in biogenesis of the microRNAs miR-30b and miR-378 and an increase of intracellular cAMP levels, both of which promote the transcriptional activation of brown adipogenesis, including UCP1 induction. The UCP1-inducing effects of EPA are also reported to be mediated via inhibition of production of ω6-derived oxygenated metabolites, such as oxylipins, that can impair UCP1 activation and induction (97). Despite the abundance of evidence in rodents, however, the thermogenic effect of EPA and DHA and its relation to BAT in humans remain to be investigated. In this context, one interesting development is a recent report by Leiria et al. (98), who observed that administration of a β3AR agonist induces a rapid increase in the plasma levels of 12- hydroxyeicosapentaenoic acid (12-HEPE) and 14-hydroxydocosahesanoic acids (14-HDHA), lipoxygenase products of EPA and DHA, in parallel with the BAT activity assessed by FDG-PET/CT, in humans. They also demonstrated in mice that activated brown adipocytes released 12-HEPE to promote glucose uptake into skeletal muscle and adipose tissues. Thus, it seems possible that 12-HEPE is a BAT-derived factor that improves insulin sensitivity and glucose metabolism (21–24).

## Bat Thermogenesis Induced by Capsaicin and Capsinoids

BAT thermogenesis is also induced by various non-caloric food ingredients and natural substances. One example of such ingredients is capsaicin, the major pungent component of chili peppers, which happens to be a potent activator of TRPV1. Capsaicin is the most consumed spice in the world, and its health beneficial effects, including thermogenic and antiobesity activities, have been known for centuries (99–101). However, because of its strong pungency, not everyone can consume capsaicin in large quantities. Capsinoids (capsiate, dihydrocapsiate, and nordihydrocapsiate) are capsaicin-like compounds found in a non-pungent type of red pepper, “CH-19 Sweet” (102). Capsaicin and capsinoids bind to TRPV1 with comparable affinities; however, pungency is much less defined in capsinoids (1/1,000). The low pungency exhibited by capsinoids may be attributable to the high lipophilicity of capsinoids, which render these molecules unable to access the termini of trigeminal nerves in the oral cavity, which is covered with epithelium (103).

Animal studies have demonstrated that oral administration of capsaicin and/or capsinoids can activate TRPV1 expressed in sensory nerves within the gastrointestinal tract and increase sympathetic nerve activity innervating BAT, inducing a rapid increase in BAT temperature, increasing whole-body EE, and decreasing body fat (104–106). These responses are blunted by the administration of β-adrenergic blockers (106) or through the denervation of vagal afferents and extrinsic nerves connected to the jejunum (104, 105). It was also reported that the thermogenic and fat-reducing effects of capsinoids are diminished in mice lacking either TRPV1 or UCP1 (105, 107, 108). Taken together, oral administration of either pungent capsaicin or non-pungent capsinoids increases whole-body EE and prevents obesity through the activation of the TRPV1–SNS–BAT axis in small rodents. It is possible that capsaicin also acts directly on TRPV1 expressed in BAT (109). By contrast, the direct action of capsinoids on TRPV1 in brown adipocytes is unlikely because orally ingested capsinoids are rapidly hydrolyzed in the small intestine and are usually undetectable in general circulation (110).

In humans, our team (111) found that a single oral ingestion of capsinoids increases EE in individuals with metabolically active BAT, but not in those without it. These findings indicate that the thermogenic effects of capsinoids are dependent on the presence of BAT—implying that capsinoids activate BAT and thereby increase EE. Furthermore, daily ingestion of capsinoids for 6 weeks augments CIT in individuals with low BAT activities (18). Because interindividual (26) and intraindividual variations of CIT (112) are significantly related to BAT activity assessed by FDG-PET/CT, the capsinoid-induced increase in CIT reflects the recruitment of BAT. This was directly confirmed by using FDG-PET/CT (113, 114) and also through near-infrared time-resolved spectroscopy (NIRTRS) (114), a novel method for evaluating BAT density in a specific region of interest (115). Thus, capsinoids have the potential to activate and recruit human BAT, thereby contributing to their antiobesity effect. Indeed, after a 12-week oral ingestion of capsinoids, a slight but significant reduction of abdominal fat was observed in a group of obese individuals (116). Notably, the fat-reducing effect of capsinoids is attenuated in individuals who carry a mutated (Val585Ile) TRPV1 (116), consistent with the crucial role of TRPV1 in mice. As single and daily oral ingestions of capsinoids at doses of 30 (110) and 6–9 mg/day for 6–12 weeks (18, 114, 116), respectively, produced no serious adverse events, dietary supplementation with capsinoids appears to be safe and feasible for combating obesity.

Although the effects of capsinoids are similar to those of cold exposure, TRPV1 is not a cold sensor, but rather a sensor of noxious hot temperatures and low pH (117). It is therefore expected that human BAT is activated by nociceptive stimuli, including TRPV1 activation. In agreement with this concept, chronic adrenergic stress induced by burn trauma results in browning of white adipose tissue (118). Hence, it is conceivable that oral ingestion of capsinoids would lead to the activation of BAT thermogenesis through the TRPV1-mediated pathway in humans, whereas cold exposure would be more potent in inducing BAT activation than capsinoid ingestion (113). In light of a recent report claiming that capsinoid treatment in mice potentiates cold-induced browning of white fat (119), a combination of capsinoid supplementation and mild cold exposure may be an effective strategy for recruitment of BAT in humans.

## Activation and Recruitment of Bat by Tea Caffeine and Catechins

Other intriguing food ingredients that activate BAT thermogenesis are caffeine and catechins, which are abundantly found in green tea. Tea is made from the leaves of *Camellia sinensis* L., a species belonging to the Theaceae family. The manufacturing process produces various types of tea such as non-oxidized, non-fermented green tea, semifermented Oolong tea, and fermented black and red teas. These teas, particularly green tea, contain relatively large amounts of polyphenols, such as epicatechin and epigallocatechin gallate, which have various health benefits such as antiobesity, anticarcinogenic, and antibacterial properties (120, 121).

An apparent thermogenic effect of green tea extract in humans was reported first by Dulloo et al. (122). They demonstrated that ingestion of green tea extract containing catechins and caffeine elicited a 4% increase in 24-h EE coupled with an increase in fat oxidation. Ingestion of caffeine alone had only a very slight effect on EE, implying that the effects of green tea extract is mainly attributable to thermogenesis by catechins. Since then, the short-term thermogenic effects of green tea extract and/or catechins have been confirmed by several studies using various doses of the extract and duration (123–125). Their long-term effects on body fatness have also been repeatedly investigated. For example, Nagao et al. (126) reported a small (2–3%) but significant reduction of body fat content in a group of Japanese volunteers who underwent 12 weeks of treatment with green tea extract containing catechins. Similar fat-reducing effects of catechins were also confirmed in other studies (127–130), although there is also a report showing that there was no significant effect (131).

Although the possible involvement of BAT in the thermogenic and antiobesity effects of catechins has been suggested (132, 133), no evidence supporting this claim has been reported in humans. Recently, we found that an oral ingestion of catechin-rich tea produced a rapid increase in EE in individuals with higher BAT activities, but not in those with undetectable BAT activities (134). Moreover, a 5-week daily ingestion of catechin-rich tea resulted in a significant increase in CIT, an index of BAT activity. Although the active and placebo beverages contained a moderate amount of caffeine, the placebo ingestion did not produce any change in EE and CIT. The chronic effects of catechins on BAT were also confirmed using the NIRSTRS technique (135). Thus, it is highly likely that the observed thermogenesis is attributable to catechin, rather than caffeine. However, this does not rule out a possible synergistic action between catechins and caffeine (136, 137). Collectively, the thermogenic and fat-reducing effects of green tea extract rich in catechins would be attributable to the activation of BAT.

The thermogenic response to green tea extract has been proposed to be mediated through the direct stimulation of the NA–βAR cascade in BAT by inhibiting a catecholamine-degrading enzyme, catechol-*O*-methyl transferase (COMT), by catechins and a cAMP-degrading enzyme, phosphodiesterase, by caffeine (133). In support of this claim, Velickovic et al. (138) demonstrated a temperature increase in the supraclavicular region, which colocates to the main region of BAT, after intake of caffeine-rich coffee. However, COMT activity may not be impaired by oral catechin ingestion in humans (139), because of the much lower circulating levels of catechins after a single ingestion (~0.1 μM at maximum) (140) compared with the half-maximal inhibitory concentration for the COMT activity (~14 μM) (141). Thus, the role of COMT inhibition as a primary target of the catechin action on BAT thermogenesis remains controversial. To this end, it is interesting that Kurogi et al. (142, 143) reported that green tea epigallocatechin gallate and its auto-oxidation products activated TRPV1 and TRP ankyrin subfamily member 1 (TRPA1), another member of the TRP family, in intestinal enteroendocrine cells at concentrations comparable to those in the gastrointestinal tract after oral ingestion. It is thus possible that catechins activate and recruit BAT through the action on TRPV1/TRPA1 in sensory neurons in the gastrointestinal tract, in the same manner as capsinoids; however, further studies are necessary to confirmthis theory.

## The TRP–Bat Axis as a Target of Antiobesity Food Ingredients

In addition to capsaicin, capsinoids, and catechins, there are other food ingredients, particularly those in spicy foods, with agonistic activity to TRPV1 (144). For example, piperine is responsible for the pungency of black and white pepper; meanwhile, gingerols, shogaol, zingerone, and 6-paradol are found in ginger, some of which might be expected to activate BAT thermogenesis and reduce body fat. The seeds of Grains of Paradise [*Aframomumu melegueta* [Rosco] *K. Schum*.] (GP), which is also known as Guinea pepper or alligator pepper, are rich in 6-paradol and are commonly used as a spice for flavoring food; they also have a wide range of ethnobotanical uses (145). In humans, we found thermogenic responses to oral ingestion of an alcohol extract of GP in individuals with metabolically active BAT, but not in those without it (146), implying a BAT-dependent thermogenesis by GP extract. In line with the acute effects, in one study, daily ingestion of GP extract for 4 weeks resulted in a slight reduction in visceral fat (147). These results suggest that GP, like capsinoids and catechins, increases whole-body EE through the activation of BAT, thereby decreasing body fatness.

As noted above, TRPV1 is not a cold sensor, but a sensor of noxious hot temperatures higher than 43°C. Among the members of the TRP family, TRP metastatin 8 (TRPM8) and TRPA1 are the most likely receptor candidates to be sensitive to lower temperatures. As the mean activation temperatures of these two TRPs are lower than 17–25°C, chemical activation of these receptors is expected to mimic the effects of a mild cold exposure. A representative TRPM8 agonist is menthol, a cooling and flavor compound in mint. Application of menthol to the skin of whole trunk in mice was shown to induce autonomic and BAT responses, but at a much lower extent in TRPM8-deficient mice (148). A rapid increase in core and BAT temperatures was also observed after intragastric administration of menthol and 1,8-cineole, another TRPM8 agonist (149). Using mice lacking either TRPM8 or UCP1, Ma et al. (150) reported that a diet supplemented with menthol enhances UCP1-dependent thermogenesis and prevents high-fat diet-induced obesity in a TRPM8-dependent manner. In humans, a slight but significant elevation of metabolic rate after a single skin menthol administration was observed (151), although its relation to BAT was not investigated.

TRPA1 is activated by various pungent compounds, such as ally- and benzyl-isothiocyanates in mustard and wasabi (Japanese horseradish) and cinnamaldehyde in cinnamon or dried bark of cassia. These compounds are known to increase thermogenesis and UCP1 expression, and decrease body fat (149, 152, 153). In addition to these food ingredients, there are various natural compounds having agonistic activity for TRPM8 and TRPA1, some of which may also have the potential to activate BAT thermogenesis and reduce body fat. However, despite the evidence for BAT activation by these food ingredients in small rodents, their thermogenic and antiobesity effects, particularly those on BAT, have yet to be elucidated in humans.

## Conclusion and Perspective

Since the rediscovery of metabolically active BAT in adult humans, BAT has been attracting increasing attention as a promising target for combating obesity and related diseases. In fact, several drugs targeting BAT have been tested for pharmacotherapy of obesity (154). In physiological terms, BAT thermogenesis is activated either by exposure to cold temperatures or after meal intake, but diet-induced BAT activation may contribute more significantly to whole-body EE in our usual daily life. As discussed above, BAT is activated by various postprandially secreted humoral factors such as BA and gut hormones, and by certain food ingredients acting on the TRP–SNS axis. Recent studies have shown that BAT is also involved in the regulation of systemic glucose and lipid homeostasis, directly by its intrinsic metabolic activity and probably through some BAT-derived humoral factors “batokines” (155). This may explain why some TRP agonists including capsinoids ameliorate insulin sensitivity and glucose homeostasis (156). Given the beneficial effects of various food ingredients and BA on substrate and energy metabolism, compounds activating the TRP/TGR5–BAT axis, by themselves and/or in combination with some drug, represent a promising option for combating obesity and related metabolic disorders.

In addition to the food ingredients discussed above, various food compounds such as curcumin, quercetin, thyme, allicin, retinoid acid, and resveratrols have been reported to activate and recruit BAT thermogenesis via multiple actions of mechanism that are either similar to or distinct from TRP-mediated processes (157, 158). Interestingly, the effects of some of these compounds including EPA are suggested to be mediated through the production of microRNAs (96, 159). However, most of these effects have been observed in studies using cells *in vitro* and mice/rats *in vivo*, whereas comparative evidence in humans is very limited. One of the reasons for this large gap may be related to the method used in assessing human BAT. To date, FDG-PET/CT is a standard tool used to measure human BAT (160); however, this option has serious limitations, including the enormous cost of the devices, radiation exposure, and acute cold exposure, which make repeated measurements difficult and an impediment in basic and clinical studies. There is therefore an urgent need to establish less invasive and simpler methods for quantitative assessment of human BAT. This would promote the development of practical, easy, and effective antiobesity regimens, particularly when searching for dietary factors/food ingredients that can activate and recruit BAT in humans.

## Author Contributions

MS wrote the first draft of the manuscript. TY reviewed the manuscript and drafted the figures. MM and YO-O critically reviewed the manuscript.

## Conflict of Interest

The authors declare that the research was conducted in the absence of any commercial or financial relationships that could be construed as a potential conflict of interest.

## Abbreviations

βAR: β-adrenergic receptor
BA: bile acids
BAT: brown adipose tissue
CCK: cholecystokinin
CIT: cold-induced thermogenesis
COMT: catechol-*O*-methyl transferase
CT: computed tomography
DIT: diet-induced thermogenesis
EE: energy expenditure
DHA: docosahexaenoic acid
EPA: eicosapentaenoic acid
FDG: fluorodeoxyglucose
GLP-1: glucagon-like peptide-1
GP: Grains of Paradise
NA: noradrenaline
NST: nonshivering metabolic thermogenesis
PET: positron emission tomography
SCTR: secretin receptor
SNS: sympathetic nervous system
TGR5: G-protein-coupled bile acid-activated receptor
TRP: transient receptor potential channel
TRPA1: TRP ankyrin subfamily member 1
TRPM8: TRP metastatin 8
TRPV1: TRP vanilloid 1
UCP1: uncoupling protein 1.
